# The association between vincristine‐induced peripheral neuropathy and health‐related quality of life in children with cancer

**DOI:** 10.1002/cam4.4289

**Published:** 2021-11-01

**Authors:** Mirjam E. van de Velde, Marleen. H. van den Berg, Gertjan J. L. Kaspers, Floor C. H. Abbink, Jos W. R. Twisk, Inge M. van der Sluis, Cor van den Bos, Marry M. van den Heuvel‐Eibrink, Heidi Segers, Christophe Chantrain, Jutte van der Werff Ten Bosch, Leen Willems, Raphaële R. L. van Litsenburg

**Affiliations:** ^1^ Emma Children’s Hospital Amsterdam UMC Vrije Universiteit Amsterdam Pediatric oncology Amsterdam The Netherlands; ^2^ Princess Máxima Center for Pediatric Oncology Utrecht The Netherlands; ^3^ Emma Children’s Hospital Amsterdam UMC Amsterdam Medical Center Pediatric Oncology Amsterdam The Netherlands; ^4^ Department of Epidemiology and Biostatistics Amsterdam UMC Vrije Universiteit Amsterdam Amsterdam The Netherlands; ^5^ Department of Pediatric Hemato‐Oncology UZ Leuven Leuven Belgium; ^6^ Department of Pediatrics Clinique MontLégia CHC Liège Belgium; ^7^ Department of Pediatric Onco‐Hematology Universitair Ziekenhuis Brussel Brussels Belgium; ^8^ Department of Paediatric Haematology‐Oncology and Stem Cell Transplantation Ghent University Hospital Ghent Belgium

**Keywords:** cancer, children, neurotoxicity, PedsQL

## Abstract

**Purpose:**

Vincristine (VCR) is a chemotherapeutic agent used in the treatment of pediatric oncology patients, but its main toxicity is VCR‐induced peripheral neuropathy (VIPN). However, whether VIPN has an effect on health‐related quality of life (HR‐QoL) in children during treatment is unknown. Therefore, the aim of our study was to investigate the association between VIPN and HR‐QoL in children starting treatment for cancer.

**Methods:**

Measurements of VIPN were performed using two tools: Common Terminology Criteria for Adverse Events (CTCAE) and pediatric‐modified Total Neuropathy Score (ped‐mTNS). Assessment of HR‐QoL was done with self‐ and proxy assessment of the Cancer and Generic module of the Pediatric Cancer Quality of Life Inventory™ (PedsQL).

**Results:**

In total, *N* = 86 children were included. HR‐QoL of children with VIPN (*n* = 67%, 76%) was significantly lower in comparison with children without VIPN: estimated Total score of PedsQL Generic (proxy) 84.57; *β* = −8.96 and 95% confidence interval (CI) −14.48 to −3.43; *p* = 0.002, estimated PedsQL Generic Total score (self‐reported): 85.16, *β* = −8.38 (95% CI: −13.76 to −3.00); *p* = 0.003. Similar results were found in the Pain and Hurt domain of the PedsQL Cancer (pain: estimated score [proxy]: 85.28, *β* = −9.94 [95%CI: −16.44 to −3.45], *p* = 0.003; hurt: estimated score [self‐report] 97.57, *β* = −19.15 [95%CI: −26.82 to −11.48], *p* < 0.001).

**Conclusion:**

VIPN results in a significant reduction of HR‐QoL in children under treatment for a malignancy, which means that VIPN is important for the well‐being of pediatric oncology patients. Therefore, this study underlines the importance of optimizing treatment with VCR, thereby aiming to reduce VIPN while maintaining efficacy.

## Graphical abstract

This article reports on the association between vincristine‐induced peripheral neuropathy and health‐related quality of life in pediatric oncology patients. Our data show that health‐related quality of life is significantly lower in children who experience vincristine‐induced peripheral neuropathy compared to pediatric oncology patients without vincristine‐induced peripheral neuropathy.

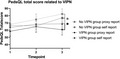

## 1 INTRODUCTION

Since the 1950s, survival of pediatric cancer gradually increased with currently an overall 5‐year survival rate of approximately 80%.^1^ However, 25% of this population experiences a severe or life‐threatening disability related to the oncologic treatment.^2^ Besides the increased incidence of disabilities, the treatment of a malignancy in childhood also leads to a reduction in health‐related quality of life (HR‐QoL) compared to healthy peers and siblings.^3^ Moreover, this reduced HR‐QoL is found in pediatric patients during anticancer treatment, but also in survivors of childhood cancer.^4^

HR‐QoL constitutes of effect of illness, treatment, and health on important domains of life, such as physical, psychological, and social domains.^5^, ^6^ Reduced HR‐QoL during anticancer treatment in children has been reported in all major domains.^7^, ^8^ Risk factors for impaired HR‐QoL in childhood cancer include (mal‐)adjustment to chronic illness in children, which can be explained by the disability‐stress‐coping model.^9^ This model describes risk‐ and protective factors that influence the ability to adjust to a chronic disease.^9^ Furthermore, HR‐QoL is related to diagnosis.^10^ Finally, HR‐QoL depends on co‐treatment of specific agents, such as glucocorticoids.^11^

Vincristine (VCR) is a chemotherapeutic agent that is often used in children with cancer. Unfortunately, VCR‐induced peripheral neuropathy (VIPN) leads to limitations in the maximum tolerable dose. Symptoms include paresthesia, constipation, muscle weakness, areflexia, neuropathic pain, and loss of sensibility.^12^ The development of VIPN during treatment depends on several factors.^13^ Treatment is symptomatic and includes the use of analgesics such as gabapentin and amitriptyline. However, the only effective treatment option for VIPN is dose‐reduction of VCR, even though this hampers optimum treatment.^14^ Alternatively, prolongation of VCR administration duration can be used even though evidence of efficacy is scarce.^15^, ^16^

It was shown that in adults, chemotherapy‐induced peripheral neuropathy is associated with reduced HR‐QoL during and after treatment.^17^ Furthermore, we know that during cancer treatment pain has an impact on HR‐QoL.^18^ However, to what extent VIPN impacts HR‐QoL in children with cancer during treatment, is unknown.

Therefore, we studied the impact of VIPN during treatment of various types of malignancies on HR‐QoL in children during the first 12 months of treatment.

## 2 METHODS

### 2.1 Patients

This study is part of a prospective trial that studied the relation between infusion duration of VCR and the development of VIPN in pediatric oncology patients (the VINCA trial).^16^ This trial was registered in the Dutch Trial Registry (www.trialregister.nl; trial number NL4019). Participants of this trial received all their VCR administrations throughout treatment either as a push‐injection or as a 1‐h infusion. In total, 10 treatment centers in the Netherlands and Belgium (*n* = 6 in Belgium, *n* = 4 in the Netherlands) were open for patient inclusion. Patients in this study were newly diagnosed children with cancer, starting VCR therapy and were between 2 and 18 years of age.

Children with different diagnoses and corresponding treatment protocols were included in this trial. The majority were hematological patients with either acute lymphoblastic leukemia (treated according to DCOG ALL‐11 protocol,^19^ EsPhALL protocol,^20^ or EORTC‐58081‐CLG guideline^21^) or Hodgkin's lymphoma (treated according to EuroNet‐PHL‐C1 protocol^22^ or C2 protocol^23^). Furthermore, solid tumor patients were included with nephroblastoma (treated according to SIOP Wilms 2001 protocol^24^) or rhabdomyosarcoma (treated according to EpSSG RMS 2005 protocol^25^). Finally, children with the following central nervous system (CNS) tumors were included: low‐grade glioma (LGG) (treated according to SIOP‐LGG 2004^26^ protocol) and medulloblastoma (treated according to ACNS0331^27^ protocol or ACNS0332^28^ protocol). Children with CNS tumors were only eligible if at diagnosis they did not experience any sensory or motor symptoms of their limbs. Finally, due to the difficulties in assessing VIPN, patients with either pre‐existent peripheral neuropathy or mental retardation could not participate in the trial.

This trial was conducted according to the principles of the Declaration of Helsinki. Furthermore, this trial adhered to all applicable country‐specific regulatory guidelines. Before trial participation, informed consent in writing was collected from guardians or parents of all trial participants between 2 and 17 years. Furthermore, children who were aged between 12 and 17 years provided informed consent in writing themselves. The informed consent form and the study protocol received approval from the Institutional Review Board of Amsterdam UMC, location VUmc and were also approved at each of the trial sites.

### 2.2 Trial regimen

In the current report, the results are reported of data collected during the first year after diagnosis. During this year, we measured HR‐QoL and VIPN between 1 and 3 times. The number of measurements depends on the amount of VCR administrations and duration of treatment (and treatment group). On average, VIPN and HR‐QoL were assessed every 3 months.

### 2.3 Assessment of VIPN

Both VIPN and HR‐QoL were assessed simultaneously on the same day of treatment. An overview of timing of measurements per disease type is shown in Figure S2. For the measurement of VIPN, two different measurement tools were used. The CTCAE items (version 4.03)^29^ peripheral motor neuropathy (score range: 0–5), peripheral sensory neuropathy (score range: 0–5), neuralgia (score range: 0–3), and constipation (score range: 0–3) were used. These item scores were summed into a total VIPN score. Furthermore, the dichotomized definition of VIPN was an item score of ≥2. In addition, the ped‐mTNS (Dutch translation) was used for VIPN assessment.^30^ This validated instrument for assessing VIPN in children includes a questionnaire (functional, sensory, and autonomic symptoms) and a physical examination. It can be used for VIPN assessment in pediatric patients aged 5 years or older. Therefore, in our study, we only used ped‐mTNS assessment in the corresponding age group. As previously published, a total ped‐mTNS score of ≥5 indicated that the child had VIPN.^31^

### 2.4 Assessment of health‐related quality of life

Assessment of HR‐QoL was done by using the Dutch translated Generic Pediatric Cancer Quality of Life Inventory™ (PedsQL) (version 4.0) and the Acute Cancer PedsQL (version 3.0) (both 1 week recall).^32^, ^33^, ^34^ Both parent/guardian‐proxy (all children) and self‐reported (aged ≥5 years) questionnaires were used. The PedsQL generic version has 23 items, which can be summed to a total scale score. Additionally, there are two other sum‐scores: psychosocial and physical. Individual items were scored on a Likert scale (5 points). However, visual scales (3‐points) that use sad, neutral, and happy faces were used for the responses of the children aged 5–7 years group. These require an interviewer for assessment. Score range is 0–100, in which a higher scores means a better HR‐QoL. The PedsQL Acute Cancer is a reliable 27‐item multidimensional cancer‐specific questionnaire.^35^ Subscales determine problems in different important areas during cancer treatment. It was hypothesized that VIPN can lead to diminished scores on the Pain and Hurt item, but is less likely to affect the other domains that are tested with the PedsQL Cancer questionnaire. Therefore, only the Pain and Hurt domain of the PedsQL Cancer was reported. Similar to the Generic version, the PedsQL Cancer also uses Likert scales (5 points) for item scores. These scores can range from 0 to 100, with higher scores being indicative for better HR‐QoL.

### 2.5 Statistics

Normally distributed descriptive data were reported as means (standard deviation [SD]). Similarly, skewed variables of descriptive data were reported as medians (interquartile range [IQR]). Patient characteristics of patients without and with VIPN were analyzed using a chi‐square test or an independent *t*‐test. Participants were identified with VIPN if they fulfilled the CTCAE or the ped‐mTNS criteria specified above at least once during follow‐up.

Differences in HR‐QoL between participants with and without VIPN were evaluated using linear mixed model analyses of PedsQL outcomes. Subgroups were analyzed for patients who had VIPN according to either ped‐mTNS *or* to CTCAE. First, the relation between PedsQL scores and VIPN was analyzed univariately, but since VIPN and HR‐QoL are multifactorial, influenced by several factors, such as sex and age,^13^, ^36^ we also used a forward selection procedure, to study variables that potentially influence differences in HR‐QoL (PedsQL generic total score and PedsQL Cancer Pain and Hurt scale) between participants with and without VIPN. The following variables were evaluated: randomization, diagnosis, sex, age, cumulative VCR dose per m^2^, use of analgesics, time since diagnosis, and ethnicity. Analyses were done in SPSS 26.0. A *p*‐value of <0.05 was used as cutoff value for statistical significance.

## 3 RESULTS

In total, *n* = 90 patients participated, of which *n* = 86 provided data on HR‐QoL. The four patients without HR‐QoL data were either insufficient in their written language proficiency or did not respond to the distributed HR‐QoL questionnaires. Descriptive information of patients is shown in Table 1.

**TABLE 1:** Patient characteristics of the total cohort and of the subgroups with and without VIPN using common toxicity criteria of adverse events

	Total group (*n* = 86)	No VIPN (*n* = 19)	VIPN (*n* = 67)	*p* value
Age at diagnosis in years, mean (SD)	9.09 (5.12)	7.92 (5.51)	9.42 (5.00)	0.42
Sex, *n* (%)				0.40
Female	39 (45)	7 (37)	35 (52)	
Male	47 (55)	12 (63)	32 (48)	
Diagnosis, *n* (%)				0.12
ALL	56 (65)	10 (53)	46 (69)	
Hodgkin	16 (19)	3 (16)	13 (19)	
Other	14 (16)	6 (32)	8 (12)	
Ethnicity, *n* (%)				0.83
Caucasian	71 (83)	16 (84)	55 (82)	
Other	15 (17)	3 (16)	12 (18)	
Use of analgesics, *n* (%)				0.01
No	68 (79)	19 (100)	49 (73)	
Yes	18 (21)	0 (0)	18 (27)	
Time since diagnosis in days, mean (SD)	158 (145)	174 (148)	139 (141)	0.26
Mean cumulative VCR dose in mg per m^2^ (SD)	14.8 (9.82)	13.12 (10.95)	15.23 (9.54)	0.07

Abbreviations: ALL, acute lymphoblastic leukemia, VCR, vincristine; VIPN, vincristine‐induced peripheral neuropathy, SD, standard deviation.

Time since diagnosis, sex, and disease were significantly associated with VIPN outcomes and total PedsQL scores (see Table S1 and Table S2) and were included, together with randomization status, in our multivariable analyses. In contrast, cumulative dose of VCR per m^2^, use of analgesics, age, and ethnicity were not significantly associated with VIPN outcomes (Table S1). However, use of analgesics was significantly associated with the outcomes of the Pain domain of the PedsQL Cancer scores and therefore included in the multivariable model of this domain.

In total, *n* = 41 (48%) developed VIPN according to CTCAE and *n* = 52 (61%) according to ped‐mTNS. In total, *n* = 67 (78%) patients met criteria for VIPN according to ped‐mTNS or CTCAE. The ped‐mTNS cohort consisted of in total *n* = 61 children (i.e., ≥5 years). Of *n* = 61 children, *n* = 26 (43%) developed VIPN according to both CTCAE and ped‐mTNS. In total, *n* = 42 (49%) patients developed severe VIPN according to either CTCAE (*n* = 8 [9%]), ped‐mTNS (*n* = 38 out of *n* = 61 [62%]) or both (*n* = 4 [7%]) at any time point during the first year of treatment. In Table S3 the HR‐QoL scores per time point are reported.

### 3.1 Relation between PedsQL generic self and proxy assessments and VIPN

Overall, estimated PedsQL Generic Total score was 84.57 (parent‐reported) and 85.16 (self‐reported). The timing of VIPN and HR‐QoL assessments in relationship to VCR treatments are presented in Figure S2.

Results of the uncorrected association between VIPN and PedsQL scores are presented in Tables S4 and S5.

**FIGURE 1:**
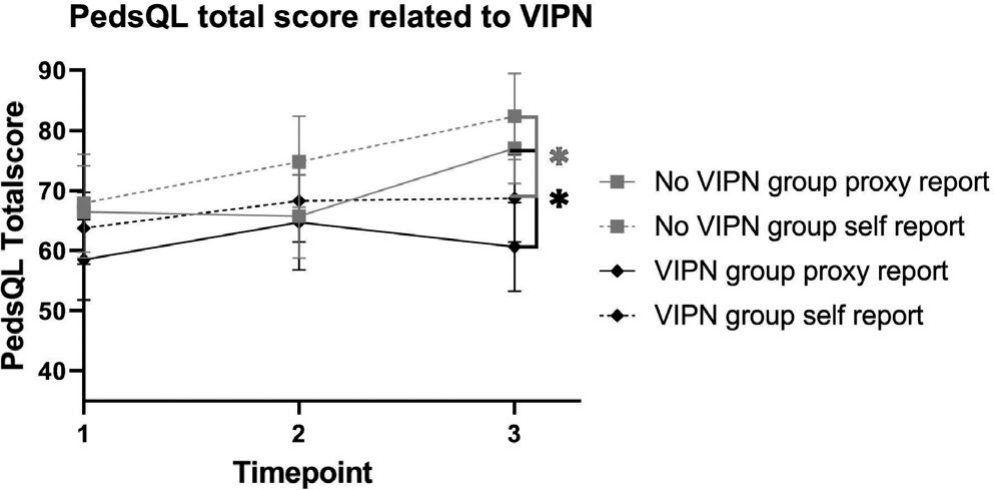
Total PedsQL generic score divided in pediatric patients with and without vincristine‐induced peripheral neuropathy according to either the common terminology criteria for adverse events or pediatric‐modified total neuropathy score. *Represents *p* values <0.05 per timepoint between the groups with and without VIPN, VIPN, vincristine‐induced peripheral neuropathy; PedsQL, Pediatric Quality of Life Inventory

There was a significantly lower HR‐QoL (proxy and self‐assessments) in children with VIPN compared to children without VIPN for both PedsQL Generic total score as well as all sub domains except School Functioning (Tables 2 and 3; Figure 1). Interestingly, in children with severe VIPN according to the ped‐mTNS, there was a significantly lower score on (proxy‐ and self‐reported) school functioning compared to children without VIPN.

**TABLE 2:** Differences in parent‐reported HR‐QoL between children with and without VIPN

	Generic total score		Generic PSHS		Generic physical functioning		Generic emotional functioning		Generic social functioning		Generic school functioning		Cancer pain	
	Beta (95% CI)	*p* value	Beta (95% CI)	*p* value	Beta (95% CI)	*p* value	Beta (95% CI)	*p* value	Beta (95% CI)	*p* value	Beta (95% CI)	*p* value	Beta (95% CI)	*p* value
VIPN according to														
CTCAE or ped‐mTNS	−8.96 (−14.48 to −3.43)	0.002	−5.95 (−11.24 to −0.67)	0.03	−13.44 (−19.48 to −7.41)	<0.001	−4.61 (−9.08 to −0.15)	0.04	−3.78 (−8.65 to 1.10)	0.13	−4.24 (−13.09 to 4.61)	0.35	−9.94 (−16.44 to −3.45)	0.003
CTCAE	−9.57 (−17.06 to −2.08)	0.01	−7.01 (−14.11 to 0.08)	0.05	−13.31 (−20.52 to −6.11)	<0.001	−7.68 (−12.84 to −2.52)	0.004	−3.36 (−9.11 to 2.40)	0.25	−6.82 (−18.56 to 4.92)	0.25	−11.70 (−19.22 to −4.19)	0.002
ped‐mTNS	−9.96 (−15.83 to −4.08)	0.001	−6.87 (−12.38 to −1.37)	0.02	−12.71 (−20.19 to −5.2)	0.001	−1.63 (−7.37 to 4.11)	0.57	−6.98 (−12.63 to −1.34)	0.02	−6.65 (−15.34 to 2.03)	0.13	−12.79 (−21.62 to −3.95)	0.005
Severe VIPN according to														
CTCAE or ped‐mTNS	−9.54 (−15.66 to −3.41)	0.003	−7.87 (−13.66 to −2.09)	0.008	−13.37 (−20.66 to −6.08)	<0.001	−7.41 (−12.76 to −2.06)	0.007	−6.86 (−12.56 to −1.16)	0.02	−8.22 (−17.94 to 1.50)	0.10	−15.27 (−22.73 to −7.82)	<0.001
CTCAE	−17.43 (−38.35 to 3.49)	0.10	−18.24 (−37.89 to 1.40)	0.07	−12.95 (−30.16 to 4.26)	0.14	−8.34 (−20.61 to 3.92)	0.18	11.16 (−2.97 to 25.29)	0.12	−16.00 (−46.95 to 14.94)	0.31	−13.21 (−29.35 to −2.93)	0.02
ped‐mTNS	−10.02 (−15.83 to −4.22)	0.001	−8.11 (−13.45 to −2.77)	0.004	−13.49 (−21.11 to −5.87)	0.001	−6.14 (−11.89 to −0.40)	0.04	−9.07 (−14.67 to −3.47)	0.002	−10.86 (−19.19 to −2.52)	0.01	−16.99 (−25.60 to −8.37)	<0.001

Results represents outcomes of a multivariable model, in which outcomes are additionally corrected for randomization of administration duration (push vs. 1‐h infusions), time since diagnosis, sex and diagnosis. The Cancer Pain scale was additionally corrected for use of analgesics. No VIPN was used as reference category in all analyses.

Abbreviations: CI, confidence Interval; CTCAE, common terminology criteria of adverse events; ped‐mTNS, pediatric‐modified Total Neuropathy Score; PSHS, psychosocial health summary score; VIPN, vincristine‐induced peripheral neuropathy.

**TABLE 3:** Differences in self‐reported HR‐QoL between children with and without VIPN

	Generic total score		Generic PSHS		Generic physical functioning		Generic emotional functioning		Generic social functioning		Generic school functioning		Cancer pain	
	Beta (95% CI)	*p* value	Beta (95% CI)	*p* value	Beta (95% CI)	*p* value	Beta (95% CI)	*p* value	Beta (95% CI)	*p* value	Beta (95% CI)	*p* value	Beta (95% CI)	*p* value
VIPN according to														
CTCAE or ped‐mTNS	−8.38 (−13.76 to −3.00)	0.003	−3.38 (−8.37 to 1.62)	0.18	−17.19 (−24.45 to −9.92)	<0.001	−8.02 (−14.11 to −1.93)	0.01	−4.32 (−9.47 to 0.83)	0.10	0.56 (−7.34 to 8.45)	0.89	−19.15 (−26.82 to −11.48)	<0.001
CTCAE	−6.91 (−14.29 to 0.47)	0.07	−4.23 (−10.90 to 2.43)	0.21	−9.58 (−19.32 to 0.16)	0.05	−6.81 (−14.64 to 1.03)	0.09	−4.85 (−11.25 to 1.55)	0.14	−2.93 (−13.53 to 7.67)	0.59	−25.28 (−34.85 to −15.72)	<0.001
ped‐mTNS	−11.42 (−16.87 to −5.97)	<0.001	−6.46 (−11.57 to −1.35)	0.01	−19.75 (−27.31 to −12.19)	<0.001	−9.05 (−15.57 to −2.53)	0.007	−6.90 (−12.16 to −1.65)	0.01	−4.01 (−12.26 to 4.24)	0.34	−17.98 (−26.04 to −9.56)	<0.001
Severe VIPN according to														
CTCAE or ped‐mTNS	−10.98 (−16.41 to −5.55)	<0.001	−7.22 (−12.27 to −2.17)	0.006	−17.14 (−24.62 to −9.66)	<0.001	−10.32 (−16.52 to −4.11)	0.001	−5.50 (−10.73 to −0.26)	0.04	−8.65 (−16.69 to −0.60)	0.04	−18.41 (−26.61 to −10.20)	<0.001
CTCAE	−12.41 (−33.30 to 8.48)	0.24	−9.08 (−27.76 to 9.60)	0.34	−18.14 (−40.92 to 4.65)	0.12	−12.54 (−30.76 to 5.68)	0.18	−11.64 (−26.20 to 2.91)	0.12	−20.69 (−49.81 to 8.43)	0.16	−25.19 (−53.32 to 2.93)	0.08
ped‐mTNS	−12.24 (−17.63 to −6.84)	<0.001	−8.43 (−13.44 to −3.41)	0.001	−18.60 (−26.23 to −10.98)	<0.001	−10.34 (−16.82 to −3.86)	0.002	−7.19 (−12.42 to −1.96)	0.007	−10.81 (−18.76 to −2.87)	0.008	−19.29 (−27.64 to −10.95)	<0.001

Results represents outcomes of a multivariable model, in which outcomes are additionally corrected for randomization of administration duration (push vs. 1‐h infusions), time since diagnosis, sex, and diagnosis. The Cancer Pain scale was additionally corrected for use of analgesics. No VIPN was used as reference category in all analyses.

Abbreviations: CI, confidence Interval; CTCAE, common terminology criteria of adverse events; ped‐mTNS, pediatric‐modified Total Neuropathy Score; PSHS, psychosocial health summary score; VIPN, vincristine‐induced peripheral neuropathy.

### 3.2 Relation between PedsQL cancer self and proxy assessments and VIPN

**FIGURE 2:**
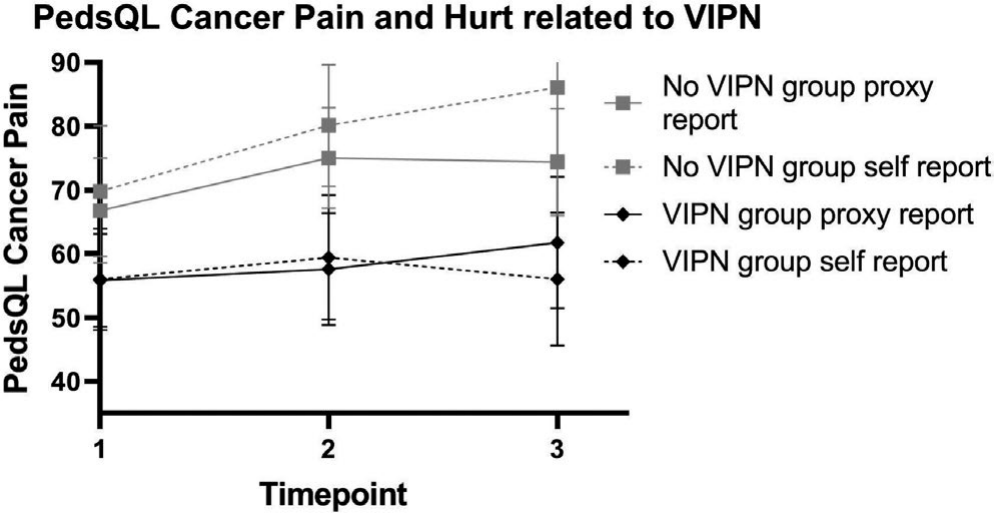
Total PedsQL Cancer scores Pain and Hurt domain divided in children with and without vincristine‐induced peripheral neuropathy according to the common terminology criteria for adverse events or pediatric‐modified total neuropathy score. All results at different time points were significant with a *p* < 0.05. VIPN, vincristine‐induced peripheral neuropathy; PedsQL, Pediatric Quality of Life Inventory

Results of the uncorrected analyses and of the univariable association with covariates are presented in Tables S4 and S5. Estimated PedsQL Cancer domain Pain and Hurt score was 85.28 (parent‐reported) and 97.57 (self‐reported). Similar to the PedsQL Generic outcomes, these and other outcomes of PedsQL Cancer Pain and Hurt scores according to VIPN status per time point of other domains are presented in Figure S1. HR‐QoL according to PedsQL Cancer Pain and Hurt in proxy as well as self‐assessments were significantly lower in our multivariable model in patients with VIPN compared to children without VIPN, irrespective of VIPN measurement tool (Tables 2 and 3). In patients with severe VIPN measured by ped‐mTNS there was a significant lower HR‐QoL compared to children without severe VIPN. However, the results of self‐assessment were not statistically significant when only the CTCAE was taken into consideration, but the estimated difference in PedsQL score in children with and without VIPN according to the CTCAE (β) was −25.19 (Tables 2 and 3; Figure 2). PedsQL Cancer scores per time point, subdivided according to VIPN status, are presented in Figure 2.

## 4 DISCUSSION

This study shows that pediatric oncology patients with VIPN have a significantly reduced HR‐QoL compared to patients without VIPN. This finding is consistent, as it was found irrespective of method of assessment of VIPN (CTCAE or ped‐mTNS), and both in self‐ as well as in proxy assessments, and on multiple HR‐QoL domains.

VCR is an important part of many pediatric oncology treatment protocols, but VIPN is a severe toxicity with symptoms that can last long after treatment cessation.^37^, ^38^ Even though VCR has been used for decades in children and its association with VIPN is well known, as far as we know, this trial is the first assessing the consequences of VIPN on HR‐QoL in children currently treated for childhood cancer. However, the effect of VIPN on HR‐QoL in pediatric oncology *survivors* was previously reported. The study of Tay et al.^38^ investigated 101 survivors of childhood ALL (mean time post‐treatment: 4.11 years) and reported a significant association between VIPN and the PedsQL, with reduced scores on the generic domains Physical Functioning and Social functioning in patients with VIPN.

Ultimately, this study shows the necessity for improving VCR treatment in children with cancer as a way to improve overall HR‐QoL of patients during oncologic treatment. Therefore, dosing of VCR should be done on a more individualized basis. For instance, it was demonstrated that certain genetic aberrations, like a single nucleotide polymorphism in centrosomal protein 72 (CEP72) influence the development of VIPN in children.^39^ Following this, studies could be undertaken that use these genetic aberrations to dose modify VCR in certain populations requiring more or less. Another approach would be to study pharmacokinetics of VCR. There is no established target VCR exposure yet, but reliable VCR PK data have been collected that could contribute to this.^13^, ^40^ When the target VCR exposure is known, VCR doses could be adapted accordingly. Finally, improving administration duration of VCR can optimize VCR treatment.^16^, ^40^, ^41^ When one of these, or other, approaches are studied and used for the optimization of VCR treatment, ideally the effect of the intervention on HR‐QoL of children should also be studied.

Finally, HR‐QoL of children is dependent on the coping and adjustment possibilities of the child and the surrounding family. Poverty and social isolation contribute to ongoing and/or escalation of distress, similar to other (pre‐existing) stressors.^42^ In general, it was demonstrated that all factors that are associated with health disparities in pediatric oncology were related to both well‐being and overall adjustment. In addition, family structure and beliefs and the natural coping ability to function are related to the adaptive adjustment to cancer (treatment). Therefore, it is important to screen for these factors in families.^43^ Interventions aimed to improve HR‐QoL in pediatric oncology patients should also take these psychosocial factors into account besides solely focusing on physical functioning.

The strengths of this trial, among others, are the comprehensive and prospective measurements of VIPN. For this study two instruments that include physical examination were used to assess VIPN at several occasions during treatment. These assessments were done using specifically trained physical therapists at all study sites. Currently, ped‐mTNS is recommended for VIPN assessment in children, as shown in a systematic review.^44^ In other studies, VIPN assessment was frequently done only by retrospective CTCAE assessment.^13^ Overall, the effects of VIPN on HR‐QoL seem to be stronger and more significant when VIPN is measured by ped‐mTNS. Previous literature showed that ped‐mTNS assessment is more sensitive in detecting VIPN than CTCAE assessment,^44^ which is supported by the fact that in our study more children with VIPN were identified by ped‐mTNS than by CTCAE. This could be an explanation for the enhanced effect of VIPN on HR‐QoL, when this was assessed by ped‐mTNS. Our data also shows that the negative effect of VIPN on HR‐QoL is stronger when VIPN is severe, even though the results of severe VIPN did not reach statistical significance for the CTCAE outcomes. This lack of significance is likely due to the low patient numbers with severe VIPN according to the CTCAE (*n* = 8).

Our study had some limitations. First of all, our results rely partly on HR‐QoL assessment by proxy responders. It was previously shown in healthy children that there is a relation between the mental health status of a parent and the proxy ratings they score.^45^ Second, this study reports on children with different disease types and treatment phases of pediatric cancer. Treatment of different diseases and treatment phases are not uniform regarding the administered VCR doses. Even though we corrected our results for disease type and did not find an association between cumulative VCR dose per m^2^ in the relation between VIPN and HR‐QoL, differences per treatment phase could still be of importance, also due to the effect of other (chemotherapeutic) treatment modalities. De Vries et al.^11^ have shown for instance that dexamethasone treatment, which is frequently used in some treatment phases of ALL but less frequent in treatment protocols of other diseases, is significantly associated with worse HR‐QoL in children with cancer. Therefore, it would be useful to investigate the VIPN/HR‐QoL relation in a more uniform patient population, to see if this association might be more attenuated in some patients. Moreover, the measurements of VIPN were assessed by a clinician, whereas HR‐QoL was assessed by patients or proxies. This could have resulted in different perceptions regarding the occurrence of VIPN between clinicians and patients (for example: clinicians that identify VIPN based on loss of deep tendon reflexes and mild constipation and patients not reporting VIPN due to lack of sensory or motor symptoms). However, VIPN is most adequately assessed using a combination of questions and physical examination. Therefore, it would be suboptimal to use questionnaires only for assessing VIPN. This is vice versa for HR‐QoL, which is most adequately assessed using standardized questionnaires such as the PedsQL, which rely on the assessment by patients and proxies and not by clinicians. Also, in the PedsQL Cancer module Pain and Hurt, the reported painful symptoms are not necessarily referring to VIPN, but other causes of pain as well. Finally, the number of included patients in this cohort was relatively low which has limited the amount of possible confounders that could be included in the corrected analysis. With a larger sample size, the influence of other confounders such as the use of additional neurotoxic agents or the use of central nervous system‐directed therapy could be included in the analyses as well.

In conclusion, this study shows that over time VIPN is negatively associated with HR‐QoL in children currently treated for a malignancy. These findings are robust, irrespective of self‐ or proxy assessment and VIPN assessment method (CTCAE or ped‐mTNS). Even though VCR is already used for decades and in multiple diseases, these findings underline the necessity for new studies aimed at optimizing VCR treatment using interventions directed at reducing the incidence and severity of VIPN during treatment of childhood cancer. Individualized treatment of VCR, for instance by using pharmacokinetic and pharmacogenomic data, will result in a tailored exposure to VCR resulting in treatment with maximum efficacy, reduced VIPN and thereby increased HR‐QoL in pediatric oncology patients.

## CONFLICT OF INTEREST

None of the authors have a conflict of interest to declare.

## ETHICS STATEMENT

Conduction of this trial happened according to the principles of the Declaration of Helsinki. Furthermore, this trial adhered to all applicable country‐specific regulatory guidelines. The informed consent form and the study protocol received approval from the Institutional Review Board of Amsterdam UMC, location VUmc and were also approved at trial site.

## Supporting information

Fig S1

Fig S2

Table S1‐S5

## Data Availability

All data were collected specifically for this trial. Data are available upon reasonable request.
