# Supplementary material for: The association between vincristine‐induced peripheral neuropathy and health‐related quality of life in children with cancer
Source: Cancer Med. 2021 Nov 1;10(22):8172–81. doi: 10.1002/cam4.4289 (PMC8607258; doi:10.1002/cam4.4289)
Supplement: Supplementary file 2 — Fig S2 [file CAM4-10-8172-s003.zip › New folder/cam44289-sup-0002-FigS2_1.pdf]

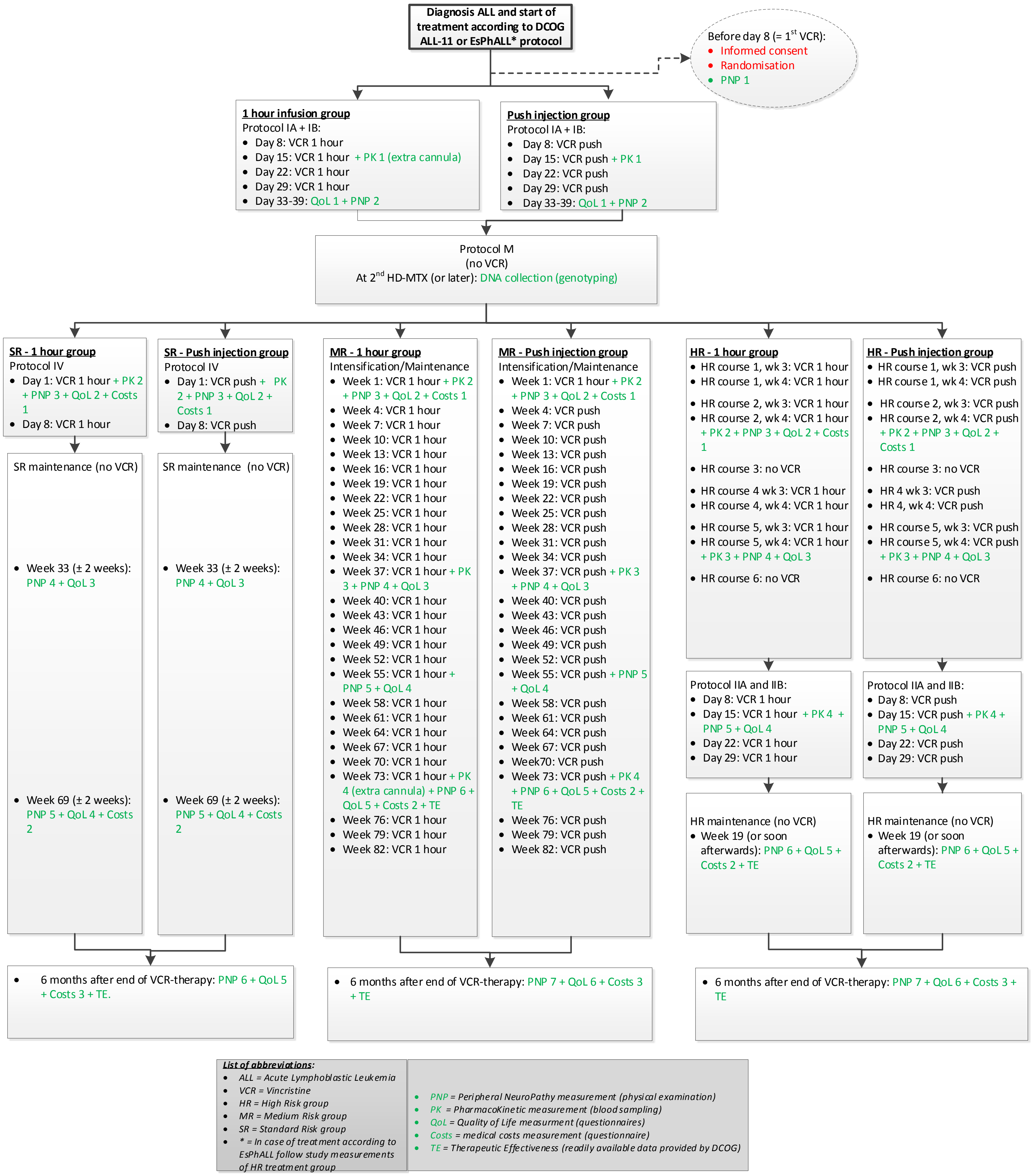
Diagnosis ALL and start of treatment according to DCOG ALL-11 or EsPhALL* protocol
Before day 8 (= 1st VCR):
Informed consent
Randomisation
PNP 1
1 hour infusion group
Protocol IA + IB:
Day 8: VCR 1 hour
Day 15: VCR 1 hour + PK 1 (extra cannula)
Day 22: VCR 1 hour
Day 29: VCR 1 hour
Day 33-39: QoL 1 + PNP 2
Push injection group
Protocol IA + IB:
Day 8: VCR push
Day 15: VCR push + PK 1
Day 22: VCR push
Day 29: VCR push
Day 33-39: QoL 1 + PNP 2
Protocol M
(no VCR)
At 2nd HD-MTX (or later): DNA collection (genotyping)
SR - 1 hour group
Protocol IV
Day 1: VCR 1 hour + PK 2 + PNP 3 + QoL 2 + Costs 1
Day 8: VCR 1 hour
SR maintenance (no VCR)
Week 33 (± 2 weeks): PNP 4 + QoL 3
Week 69 (± 2 weeks): PNP 5 + QoL 4 + Costs 2
SR - Push injection group
Protocol IV
Day 1: VCR push + PK 2 + PNP 3 + QoL 2 + Costs 1
Day 8: VCR push
SR maintenance (no VCR)
Week 33 (± 2 weeks): PNP 4 + QoL 3
Week 69 (± 2 weeks): PNP 5 + QoL 4 + Costs 2
MR - 1 hour group
Intensification/Maintenance
Week 1: VCR 1 hour + PK 2 + PNP 3 + QoL 2 + Costs 1
Week 4: VCR 1 hour
Week 7: VCR 1 hour
Week 10: VCR 1 hour
Week 13: VCR 1 hour
Week 16: VCR 1 hour
Week 19: VCR 1 hour
Week 22: VCR 1 hour
Week 25: VCR 1 hour
Week 28: VCR 1 hour
Week 31: VCR 1 hour
Week 34: VCR 1 hour
Week 37: VCR 1 hour + PK 3 + PNP 4 + QoL 3
Week 40: VCR 1 hour
Week 43: VCR 1 hour
Week 46: VCR 1 hour
Week 49: VCR 1 hour
Week 52: VCR 1 hour
Week 55: VCR 1 hour + PNP 5 + QoL 4
Week 58: VCR 1 hour
Week 61: VCR 1 hour
Week 64: VCR 1 hour
Week 67: VCR 1 hour
Week 70: VCR 1 hour
Week 73: VCR 1 hour + PK 4 (extra cannula) + PNP 6 + QoL 5 + Costs 2 + TE
Week 76: VCR 1 hour
Week 79: VCR 1 hour
Week 82: VCR 1 hour
MR - Push injection group
Intensification/Maintenance
Week 1: VCR 1 hour + PK 2 + PNP 3 + QoL 2 + Costs 1
Week 4: VCR push
Week 7: VCR push
Week 10: VCR push
Week 13: VCR push
Week 16: VCR push
Week 19: VCR push
Week 22: VCR push
Week 25: VCR push
Week 28: VCR push
Week 31: VCR push
Week 34: VCR push
Week 37: VCR push + PK 3 + PNP 4 + QoL 3
Week 40: VCR push
Week 43: VCR push
Week 46: VCR push
Week 49: VCR push
Week 52: VCR push
Week 55: VCR push + PNP 5 + QoL 4
Week 58: VCR push
Week 61: VCR push
Week 64: VCR push
Week 67: VCR push
Week70: VCR push
Week 73: VCR push + PK 4 + PNP 6 + QoL 5 + Costs 2 + TE
Week 76: VCR push
Week 79: VCR push
Week 82: VCR push
HR - 1 hour group
HR course 1, wk 3: VCR 1 hour
HR course 1, wk 4: VCR 1 hour
HR course 2, wk 3: VCR 1 hour
HR course 2, wk 4: VCR 1 hour + PK 2 + PNP 3 + QoL 2 + Costs 1
HR course 3: no VCR
HR course 4 wk 3: VCR 1 hour
HR course 4, wk 4: VCR 1 hour
HR course 5, wk 3: VCR 1 hour
HR course 5, wk 4: VCR 1 hour + PK 3 + PNP 4 + QoL 3
HR course 6: no VCR
Protocol IIA and IIB:
Day 8: VCR 1 hour
Day 15: VCR 1 hour + PK 4 + PNP 5 + QoL 4
Day 22: VCR 1 hour
Day 29: VCR 1 hour
HR maintenance (no VCR)
Week 19 (or soon afterwards): PNP 6 + QoL 5 + Costs 2 + TE
HR - Push injection group
HR course 1, wk 3: VCR push
HR course 1, wk 4: VCR push
HR course 2, wk 3: VCR push
HR course 2, wk 4: VCR push + PK 2 + PNP 3 + QoL 2 + Costs 1
HR course 3: no VCR
HR 4 wk 3: VCR push
HR 4, wk 4: VCR push
HR course 5, wk 3: VCR push
HR course 5, wk 4: VCR push + PK 3 + PNP 4 + QoL 3
HR course 6: no VCR
Protocol IIA and IIB:
Day 8: VCR push
Day 15: VCR push + PK 4 + PNP 5 + QoL 4
Day 22: VCR push
Day 29: VCR push
HR maintenance (no VCR)
Week 19 (or soon afterwards): PNP 6 + QoL 5 + Costs 2 + TE
6 months after end of VCR-therapy: PNP 6 + QoL 5 + Costs 3 + TE.
6 months after end of VCR-therapy: PNP 7 + QoL 6 + Costs 3 + TE
6 months after end of VCR-therapy: PNP 7 + QoL 6 + Costs 3 + TE
List of abbreviations:
ALL = Acute Lymphoblastic Leukemia
VCR = Vincristine
HR = High Risk group
MR = Medium Risk group
SR = Standard Risk group
* = In case of treatment according to EsPhALL follow study measurements of HR treatment group
PNP = Peripheral NeuroPathy measurement (physical examination)
PK = PharmacoKinetic measurement (blood sampling)
QoL = Quality of Life measurment (questionnaires)
Costs = medical costs measurement (questionnaire)
TE = Therapeutic Effectiveness (readily available data provided by DCOG)
