# Supplementary material for: The association between vincristine‐induced peripheral neuropathy and health‐related quality of life in children with cancer
Source: Cancer Med. 2021 Nov 1;10(22):8172–81. doi: 10.1002/cam4.4289 (PMC8607258; doi:10.1002/cam4.4289)
Supplement: Supplementary file 2 — Fig S2 [file CAM4-10-8172-s003.zip › New folder/cam44289-sup-0003-FigS2_2.pdf]

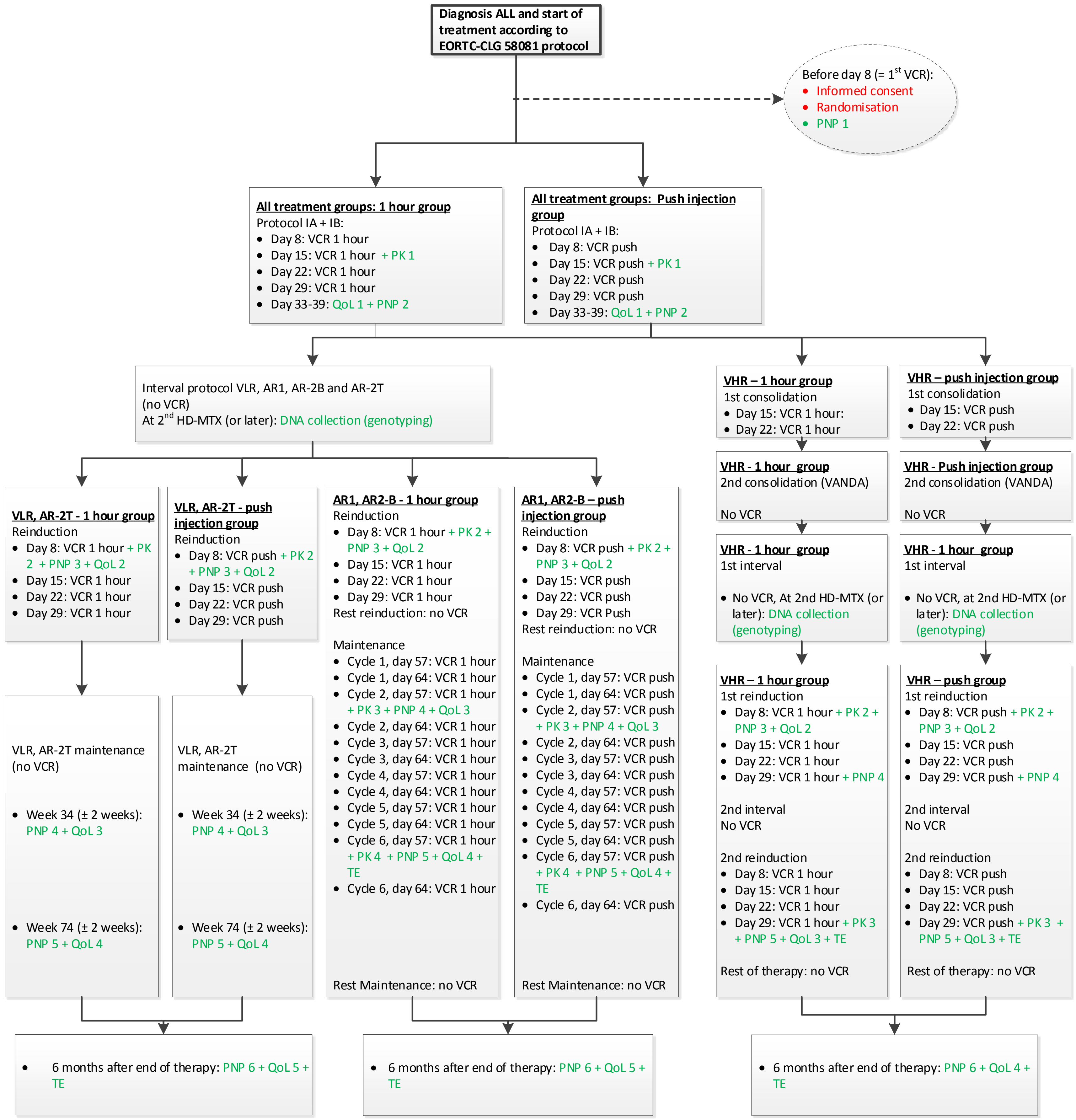

List of abbreviations:

- *ALL = Acute Lymphoblastic Leukemia*
- *VCR = Vincristine*
- *VLR = Very Low Risk group*
- *AR-B = Average Risk B cell group*
- *AR-T = Average Risk T cell group*
- *VHR = Very High Risk group*

- *PNP = Peripheral NeuroPathy measurement (physical examination)*
- *PK = PharmacoKinetic measurement (blood sampling)*
- *QoL = Quality of Life measurment (questionnaires)*
- *Costs = medical costs measurement (questionnaire)*
- *TE = Therapeutic Effectiveness (readily available data provided by DCOG)*
