# Supplementary material for: The association between vincristine‐induced peripheral neuropathy and health‐related quality of life in children with cancer
Source: Cancer Med. 2021 Nov 1;10(22):8172–81. doi: 10.1002/cam4.4289 (PMC8607258; doi:10.1002/cam4.4289)
Supplement: Supplementary file 2 — Fig S2 [file CAM4-10-8172-s003.zip › New folder/cam44289-sup-0005-FigS2_4.pdf]

Diagnosis LGG and start of chemotherapy treatment according to SIOP LGG 2004 protocol

Before day 1 (= 1st VCR):
- Informed consent
- Randomisation
- PNP 1

1 hour infusion group

Protocol induction:
- Week 1: VCR 1 hour
- Week 2: VCR 1 hour + PK 1
- Week 3: VCR 1 hour
- Week 4: VCR 1 hour + QoL 1 + PNP 2
- Week 5: VCR 1 hour
- Week 6: VCR 1 hour
- Week 7: VCR 1 hour
- Week 8: VCR 1 hour
- Week 9: VCR 1 hour
- Week 10: VCR 1 hour + PK 2 + PNP 3 + QoL 2 + Costs 1
- Week 13: VCR 1 hour
- Week 17: VCR 1 hour
- Week 21: VCR 1 hour

Push injection group

Protocol induction:
- Week 1: VCR push
- Week 2: VCR push + PK 1
- Week 3: VCR push
- Week 4: VCR push + QoL 1 + PNP 2
- Week 5: VCR push
- Week 6: VCR push
- Week 7: VCR push
- Week 8: VCR push
- Week 9: VCR push
- Week 10: VCR push + PK 2 + PNP 3 + QoL 2 + Costs 1
- Week 13: VCR push
- Week 17: VCR push
- Week 21: VCR push

Treatment response phase
(no VCR)
Week 23: DNA collection (genotyping)

Consolidation - 1 hour group
- Week 25: VCR 1 hour
- Week 26: VCR 1 hour
- Week 27: VCR 1 hour
- Week 31: VCR 1 hour
- Week 32: VCR 1 hour
- Week 33: VCR 1 hour + PK 3 + PNP 4 + QoL 3
- Week 37: VCR 1 hour
- Week 38: VCR 1 hour
- Week 39: VCR 1 hour
- Week 43: VCR 1 hour
- Week 44: VCR 1 hour
- Week 45: VCR 1 hour
- Week 49: VCR 1 hour
- Week 50: VCR 1 hour
- Week 51: VCR 1 hour + PNP 5 + QoL 4
- Week 55: VCR 1 hour
- Week 56: VCR 1 hour
- Week 57: VCR 1 hour
- Week 61: VCR 1 hour
- Week 62: VCR 1 hour
- Week 63: VCR 1 hour
- Week 67: VCR 1 hour
- Week 68: VCR 1 hour
- Week 69: VCR 1 hour
- Week 73: VCR 1 hour
- Week 74: VCR 1 hour
- Week 75: VCR 1 hour + PK 4 + PNP 6 + QoL 5 + Costs 2 + TE
- Week 79: VCR 1 hour
- Week 80: VCR 1 hour
- Week 81: VCR 1 hour

Consolidation – Push injection group
- Week 25: VCR push
- Week 26: VCR push
- Week 27: VCR push
- Week 31: VCR push
- Week 32: VCR push
- Week 33: VCR push + PK 3 + PNP 4 + QoL 3
- Week 37: VCR push
- Week 38: VCR push
- Week 39: VCR push
- Week 43: VCR push
- Week 44: VCR push
- Week 45: VCR push
- Week 49: VCR push
- Week 50: VCR push
- Week 51: VCR push + PNP 5 + QoL 4
- Week 55: VCR push
- Week 56: VCR push
- Week 57: VCR push
- Week 61: VCR push
- Week 62: VCR push
- Week 63: VCR push
- Week 67: VCR push
- Week 68: VCR push
- Week 69: VCR push
- Week 73: VCR push
- Week 74: VCR push
- Week 75: VCR push + PK 4 + PNP 6 + QoL 5 + Costs 2 + TE
- Week 79: VCR push
- Week 80: VCR push
- Week 81: VCR push

- 6 months after end of VCR-therapy: PNP 7 + QoL 6 + Costs 3 + TE

List of abbreviations:
- *VCR = Vincristine*
- *LGG = Low Grade Glioma*
- *SIOP = International Society of Paediatric Oncology*
- *PNP = Peripheral NeuroPathy measurement (physical examination)*
- *PK = PharmacoKinetic measurement (blood sampling)*
- *QoL = Quality of Life measurment (questionnaires)*
- *Costs = medical costs measurement (questionnaire)*
- *TE = Therapeutic Effectiveness (readily available data provided by Dutch Childhood Oncology Group)*
