# Supplementary material for: The association between vincristine‐induced peripheral neuropathy and health‐related quality of life in children with cancer
Source: Cancer Med. 2021 Nov 1;10(22):8172–81. doi: 10.1002/cam4.4289 (PMC8607258; doi:10.1002/cam4.4289)
Supplement: Supplementary file 2 — Fig S2 [file CAM4-10-8172-s003.zip › New folder/cam44289-sup-0006-FigS2_5.pdf]

Diagnosis of SR Medulloblastoma and start of treatment according to ACNS0331 protocol

Before day 31 (= 1st VCR):
- Informed consent
- Randomisation
- PNP 1

1 hour infusion group

Chemoradiotherapy:
- Week 2: VCR 1 hour
- Week 3: VCR 1 hour + PK 1
- Week 4: VCR 1 hour
- Week 5: VCR 1 hour + QoL 1 + PNP 2
- Week 6: VCR 1 hour
- Week 7: VCR 1 hour

Push injection group

Chemoradiotherapy:
- Week 2: VCR push
- Week 3: VCR push + PK 1
- Week 4: VCR push
- Week 5: VCR push + QoL 1 + PNP 2
- Week 6: VCR push
- Week 7: VCR push

Rest
(no VCR)
One week into rest: DNA collection (genotyping)

1 Hour infusion group

Maintenance:
- Week 11: VCR 1 hour + PK 2 + PNP 3 + QoL 2 + Costs 1
- Week 12: VCR 1 hour
- Week 13: VCR 1 hour
- Week 17: VCR 1 hour
- Week 18: VCR 1 hour
- Week 19: VCR 1 hour
- Week 23: VCR 1 hour
- Week 24: VCR 1 hour + PK 3 + PNP 4 + QoL 3
- Week 27: VCR 1 hour
- Week 28: VCR 1 hour
- Week 29: VCR 1 hour
- Week 33: VCR 1 hour
- Week 34: VCR 1 hour
- Week 35: VCR 1 hour
- Week 39: VCR 1 hour + PNP 5 + QoL 4
- Week 40: VCR 1 hour
- Week 43: VCR 1 hour
- Week 44: VCR 1 hour
- Week 45: VCR 1 hour
- Week 49: VCR 1 hour
- Week 50: VCR 1 hour + PK 4 + PNP 6 + QoL 5 + Costs 2 + TE
- Week 51: VCR 1 hour
- Week 55: VCR 1 hour
- Week 56: VCR 1 hour

Push injection group

Maintenance:
- Week 11: VCR push + PK 2 + PNP 3 + QoL 2 + Costs 1
- Week 12: VCR push
- Week 13: VCR push
- Week 17: VCR push
- Week 18: VCR push
- Week 19: VCR push
- Week 23: VCR push
- Week 24: VCR push + PK 3 + PNP 4 + QoL 3
- Week 27: VCR push
- Week 28: VCR push
- Week 29: VCR push
- Week 33: VCR push
- Week 34: VCR push
- Week 35: VCR push
- Week 39: VCR push + PNP 5 + QoL 4
- Week 40: VCR push
- Week 43: VCR push
- Week 44: VCR push
- Week 45: VCR push
- Week 49: VCR push
- Week 50: VCR push + PK 4 + PNP 6 + QoL 5 + Costs 2 + TE
- Week 51: VCR push
- Week 55: VCR push
- Week 56: VCR push

- 6 months after end of VCR-therapy: PNP 7 + QoL 6 + Costs 3 + TE

***List of abbreviations*:**
- *VCR = Vincristine*
- *SR = Standard Risk group*
- *PNP = Peripheral NeuroPathy measurement (physical examination)*
- *PK = PharmacoKinetic measurement (blood sampling)*
- *QoL = Quality of Life measurment (questionnaires)*
- *Costs = medical costs measurement (questionnaire)*
- *TE = Therapeutic Effectiveness (readily available data provided by Dutch Childhood Oncology Group)*
