# Supplementary material for: The association between vincristine‐induced peripheral neuropathy and health‐related quality of life in children with cancer
Source: Cancer Med. 2021 Nov 1;10(22):8172–81. doi: 10.1002/cam4.4289 (PMC8607258; doi:10.1002/cam4.4289)
Supplement: Supplementary file 2 — Fig S2 [file CAM4-10-8172-s003.zip › New folder/cam44289-sup-0009-FigS2_8.pdf]

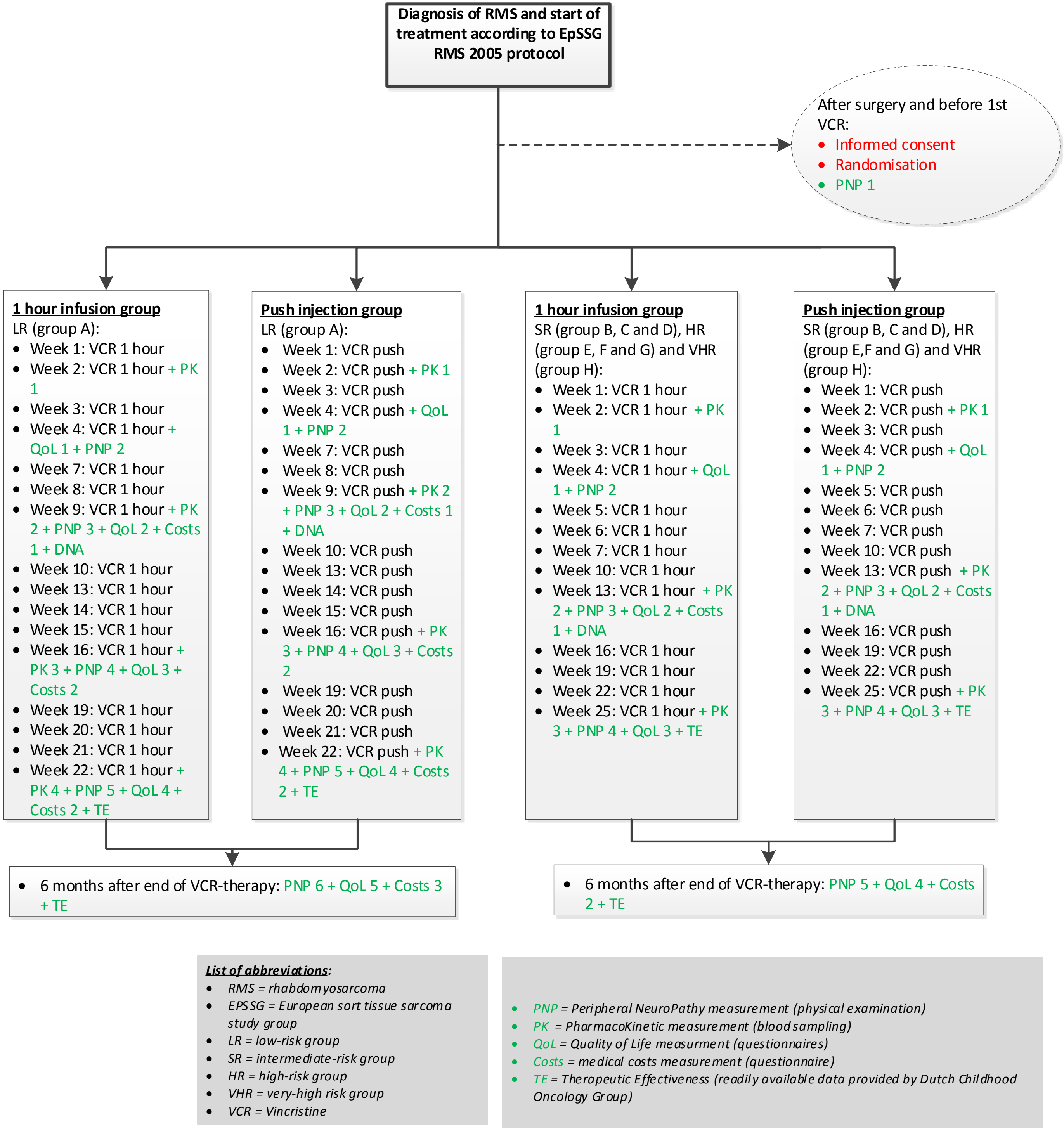
Diagnosis of RMS and start of treatment according to EpSSG RMS 2005 protocol
After surgery and before 1st VCR:
• Informed consent
• Randomisation
• PNP 1
1 hour infusion group
LR (group A):
• Week 1: VCR 1 hour
• Week 2: VCR 1 hour + PK 1
• Week 3: VCR 1 hour
• Week 4: VCR 1 hour + QoL 1 + PNP 2
• Week 7: VCR 1 hour
• Week 8: VCR 1 hour
• Week 9: VCR 1 hour + PK 2 + PNP 3 + QoL 2 + Costs 1 + DNA
• Week 10: VCR 1 hour
• Week 13: VCR 1 hour
• Week 14: VCR 1 hour
• Week 15: VCR 1 hour
• Week 16: VCR 1 hour + PK 3 + PNP 4 + QoL 3 + Costs 2
• Week 19: VCR 1 hour
• Week 20: VCR 1 hour
• Week 21: VCR 1 hour
• Week 22: VCR 1 hour + PK 4 + PNP 5 + QoL 4 + Costs 2 + TE
Push injection group
LR (group A):
• Week 1: VCR push
• Week 2: VCR push + PK 1
• Week 3: VCR push
• Week 4: VCR push + QoL 1 + PNP 2
• Week 7: VCR push
• Week 8: VCR push
• Week 9: VCR push + PK 2 + PNP 3 + QoL 2 + Costs 1 + DNA
• Week 10: VCR push
• Week 13: VCR push
• Week 14: VCR push
• Week 15: VCR push
• Week 16: VCR push + PK 3 + PNP 4 + QoL 3 + Costs 2
• Week 19: VCR push
• Week 20: VCR push
• Week 21: VCR push
• Week 22: VCR push + PK 4 + PNP 5 + QoL 4 + Costs 2 + TE
1 hour infusion group
SR (group B, C and D), HR (group E, F and G) and VHR (group H):
• Week 1: VCR 1 hour
• Week 2: VCR 1 hour + PK 1
• Week 3: VCR 1 hour
• Week 4: VCR 1 hour + QoL 1 + PNP 2
• Week 5: VCR 1 hour
• Week 6: VCR 1 hour
• Week 7: VCR 1 hour
• Week 10: VCR 1 hour
• Week 13: VCR 1 hour + PK 2 + PNP 3 + QoL 2 + Costs 1 + DNA
• Week 16: VCR 1 hour
• Week 19: VCR 1 hour
• Week 22: VCR 1 hour
• Week 25: VCR 1 hour + PK 3 + PNP 4 + QoL 3 + TE
Push injection group
SR (group B, C and D), HR (group E,F and G) and VHR (group H):
• Week 1: VCR push
• Week 2: VCR push + PK 1
• Week 3: VCR push
• Week 4: VCR push + QoL 1 + PNP 2
• Week 5: VCR push
• Week 6: VCR push
• Week 7: VCR push
• Week 10: VCR push
• Week 13: VCR push + PK 2 + PNP 3 + QoL 2 + Costs 1 + DNA
• Week 16: VCR push
• Week 19: VCR push
• Week 22: VCR push
• Week 25: VCR push + PK 3 + PNP 4 + QoL 3 + TE
• 6 months after end of VCR-therapy: PNP 6 + QoL 5 + Costs 3 + TE
• 6 months after end of VCR-therapy: PNP 5 + QoL 4 + Costs 2 + TE
List of abbreviations:
• RMS = rhabdomyosarcoma
• EPSSG = European sort tissue sarcoma study group
• LR = low-risk group
• SR = intermediate-risk group
• HR = high-risk group
• VHR = very-high risk group
• VCR = Vincristine
• PNP = Peripheral NeuroPathy measurement (physical examination)
• PK = PharmacoKinetic measurement (blood sampling)
• QoL = Quality of Life measurment (questionnaires)
• Costs = medical costs measurement (questionnaire)
• TE = Therapeutic Effectiveness (readily available data provided by Dutch Childhood Oncology Group)
