# Supplementary material for: The association between vincristine‐induced peripheral neuropathy and health‐related quality of life in children with cancer
Source: Cancer Med. 2021 Nov 1;10(22):8172–81. doi: 10.1002/cam4.4289 (PMC8607258; doi:10.1002/cam4.4289)
Supplement: Supplementary file 3 — Table S1‐S5 [file CAM4-10-8172-s002.docx]

**Supplemental Table S1** Association between parent-proxy reported PedsQL and vincristine induced peripheral neuropathy and effect of covariates

|  | PedsQL Generic Total score  Parent-proxy report | | PedsQL Generic Total score  Self-report | | PedsQL Cancer Pain parent report | | PedsQL Cancer Pain self-report | |
| --- | --- | --- | --- | --- | --- | --- | --- | --- |
|  | **Beta (95% CI)** | **P value** | **Beta (95% CI)** | **P value** | **Beta (95% CI)** | **P value** | **Beta (95% CI)** | **P value** |
| VIPN (any)  - Randomization  One-hour vs push  - Time since diagnosis (days)  - Diagnosis:  ALL vs other  Hodgkin vs other  - Sex  - Cumulative VCR dose (mg)  - Age (years)  - Use of pain medication  No analgesics vs analgesics  - Ethnicity  Caucasian vs other | 3.00 (-4.30 to 10.30)  -9.44 (-15.11 to -3.78)  -12.73 (-21.48 to -3.97)  -2.75 (-14.07 to 8.58)  -7.37 (-14.54 to -0.20)  0.18 (-0.22 to 0.57)  0.33 (-0.50 to 1.16)  5.70 (-4.03 to 15.43)  -1.03 (-10.17 to 8.11) | 0.42  0.001  0.005  0.63  0.04  0.38  0.43  0.25  0.82 | 7.39 (-0.04 to 14.81)  0.02 (0.00 to 0.04)  -9.14 (-20.32 to 2.04)  2.98 (-9.85 to 15.81)  -6.26 (-13.71 to 1.19)  0.02 (-0.53 to 0.57)  0.10 (-1.02 to 0.81)  3.63 (-5.17 to 12.45)  2.45 (-6.61 to 11.57) | 0.05  0.02  0.11  0.64  0.10  0.95  0.82  0.41  0.59 | 5.45 (-4.09 to 15.00)  0.02 (0.00 to 0.04)  -17.65 (-30.46 to -4.83)  -18.47 (-34.65 to -2.28)  -3.70 (-13.33 to 5.92)  0.28 (-0.21 to 0.77)  -0.69 (-1.66 to 0.28)  10.00 (-1.58 to 21.59)  -10.01 (-22.42 to 2.39) | 0.26  0.04  0.008  0.03  0.45  0.26  0.16  0.09  0.11 | 3.65 (-6.81 to 14.10)  0.02 (-0.00 to 0.05)  -18.71 (-35.98 to -1.43)  -19.65 (-39.11 to -0.20)  -3.69 (-14.22 to 6.84)  -0.34 (-0.46 to 1.14)  -0.58 (-1.84 to 0.69)  10.38 (-2.14 to 22.90)  -3.88 (-16.53 to 8.78) | 0.49  0.07  0.03  0.05  0.49  0.40  0.37  0.10  0.54 |
| VIPN (CTCAE)  - Randomization  One-hour vs push  - Time since diagnosis (days)  - Diagnosis:  ALL vs other  Hodgkin vs other  - Sex  - Cumulative VCR dose (mg)  - Age (years)  - Use of pain medication  No analgesics vs analgesics  - Ethnicity  Caucasian vs other | 4.29 (-2.78 to 11.36)  0.02 (0.01 to 0.04)  -12.16 (-20.69 to -3.64)  -5.10 (-16.00 to 5.80)  -6.52 (-13.75 to 0.70)  0.25 (-.12 to 0.62)  -0.05 (-0.84 to 0.74)  8.47 (-1.06 to 18.00)  -3.10 (-11.93 to 5.76) | 0.23  0.006  0.006  0.35  0.08  0.17  0.89  0.08  0.49 | 8.15 (0.79 to 15.51)  0.02 (0.00 to 0.04)  -7.37 (-18.98 to 4.24)  2.55 (-10.73 to 15.82)  -5.99 (-13.53 to 1.54)  -0.00 (-0.56 to 0.56)  -0.27 (-1.19 to 0.64)  4.80 (-4.11 to 13.70)  1.36 (-7.84 to 10.56) | 0.03  0.01  0.21  0.70  0.12  0.99  0.55  0.29  0.77 | 5.96 (-3.68 to 15.59)  0.02 (0.00 to 0.04)  -17.60 (-30.52 to -4.67)  -21.18 (-37.36 to -5.01)  -3.04 (-12.82 to 6.74)  0.35 (-0.15 to 0.85)  -1.10 )-2.04 to -0.16)  11.98 (0.61 to 23.35)  -11.51 (-24.01 to 0.98) | 0.22  0.04  0.008  0.01  0.54  0.16  0.02  0.04  0.07 | 5.16 (-4.63 to 14.94)  0.02 (-0.00 to 0.05)  -13.30 (-29.99 to 3.40)  -17.64 (-36.44 to 1.16)  -2.32 (-12.32 to 7.69)  0.15 (-0.63 to 0.93)  -0.87 (-2.06 to 0.33)  14.70 (2.38 to 27.03)  -5.96 (-17.86 to 5.93) | 0.30  0.06  0.12  0.07  0.64  0.70  0.15  0.02  0.32 |
| VIPN (ped-mTNS)  - Randomization  One-hour vs push  - Time since diagnosis (days)  - Diagnosis:  ALL vs other  Hodgkin vs other  - Sex  - Cumulative VCR dose (mg)  - Age (years)  - Use of pain medication  No analgesics vs analgesics  - Ethnicity  Caucasian vs other | 3.63 (-5.90 to 13.17)  0.02 (0.00 to 0.04)  -16.65 (-31.26 to -2.04)  -6.91 (-23.15 to 9.32)  -7.27 (-16.65 to 2.12)  0.18 (-0.54 to 0.89)  0.29 (-0.88 to 1.45)  3.61 (-5.73 to 12.95)  -1.27 (-13.00 to 10.46) | 0.45  0.02  0.03  0.39  0.13  0.62  0.62  0.44  0.83 | 7.37 (-0.30 to 15.05)  0.02 (-0.00 to 0.03)  -7.27 (-19.63 to 5.10)  4.34 (-9.65 to 18.32)  -6.94 (-14.61 to 0.72)  -0.02 (-0.63 to 0.59)  -0.18 (-1.14 to 0.77)  3.10 (-5.19 to 11.38)  2.69 (-6.78 to 12.17) | 0.06  0.07  0.24  0.54  0.08  0.95  0.70  0.46  0.57 | 4.51 (-8.30 to 17.31)  0.02 (0.01 to 0.05)  -27.74 (-50.79 to -4.68)  -31.07 to -56.41 to -5.72)  -1.11 (-14.10 to 11.86)  0.14 (-0.85 to 1.13)  -1.10 (-2.59 to 0.42)  9.64 (-3.60 to 22.87)  -9.15 (-24.95 to 6.65) | 0.48  0.11  0.02  0.02  0.86  0.78  0.15  0.15  0.25 | 1.38 (-9.57 to 12.32)  0.02 (-0.00 to 0.05)  -15.37 (-34.20 to 3.45)  -16.38 (-37.43 to 4.66)  -3.09 (-14.08 to 7.90)  0.18 (-0.68 to 1.04)  -0.72 (-2.01 to 0.57)  12.10 (-0.57 to 24.77)  -0.54 (-14.16 to 13.08) | 0.80  0.06  0.11  0.12  0.58  0.67  0.27  0.06  0.94 |

*PedsQL: Pediatric Quality of Life Inventory™, CI: confidence interval, VIPN: vincristine induced peripheral neuropathy, ALL: acute lymphoblastic leukemia, CTCAE: Common Terminology Criteria for Adverse Events, ped-mTNS: pediatric modified total neuropathy score*

**Supplemental Table S2** The association between pedsQL outcomes and randomization (VCR administrations given as either push-injection or one-hour infusion)

|  | Beta (95%CI)* | P value |
| --- | --- | --- |
| PedsQL proxyreport  Generic Totalscore  Generic PSHS  Generic Physical functioning  Generic Emotional functioning  Generic Social functioning  Generic School functioning  Cancer Pain | 3.07 (-4.54 to 10.68)  1.32 (-5.54 to 8.17)  5.08 (-4.43 to 14.61)  2.28 (-4.99 to 9.56)  0.17 (-6.17 to 6.52)  1.20 (-8.14 to 10.55)  5.19 (-5.13 to 15.51) | 0.42  0.70  0.29  0.53  0.96  0.98  0.32 |
| PedsQL selfreport  Generic Totalscore  Generic PSHS  Generic Physical functioning  Generic Emotional functioning  Generic Social functioning  Generic School functioning  Cancer Pain | 8.13 (0.48 to 15.79)  8.50 (1.95 to 15.05)  4.52 (-6.73 to 15.77)  5.33 (-3.34 to 14.00)  6.11 (-0.47 to 12.69)  11.06 (1.01 to 21.10)  5.20 (-6.50 to 16.90) | 0.04  0.01  0.42  0.22  0.07  0.03  0.38 |

*Push injections as reference; CI: confidence interval; PedsQL: Pediatric Cancer Quality of Life Inventory™; PSHS: psychosocial health summary score*

**Supplemental Table S3.** Mean Generic and Cancer PedsQL scores of proxy- and self-report per time point

|  | T=1 | | T=2 | | T=3 | |
| --- | --- | --- | --- | --- | --- | --- |
|  | **N** | **Estimated score**  **(95% CI)** | **N** | **Estimated score**  **(95% CI)** | **N** | **Estimated score**  **(95% CI)** |
| PedsQL proxy-report  Generic Total score  Generic PSHS  Generic Physical functioning  Generic Emotional functioning  Generic Social functioning  Generic School functioning  Cancer Pain | 38  38  74  75  70  38  80 | 62.24 (56.76 to 67.71)  67.11 (62.18 to 72.04)  49.11 (43.26 to 54.97)  60.86 (56.55 to 65.16)  81.39 (77.15 to 85.63)  58.93 (51.12 to 66.75)  60.63 (54.57 to 66.69) | 35  35  65  64  63  35  67 | 65.10 (59.43 to 70.78)  66.36 (61.25 to 71.47)  63.42 (57.25 to 69.60)  59.85 (55.28 to 64.42)  79.07 (74.62 to 83.52)  59.64 (51.51 to 67.78)  67.25 (60.76 to 73.74) | 47  47  53  53  53  47  52 | 70.69 (65.70 to 75.68)  71.62 (67.12 to 76.11)  68.10 (61.40 to 74.80)  68.92 (64.04 to 73.80)  80.07 (75.27 to 84.87)  67.94 (60.90 to 74.98)  68.88 (61.80 to 75.96) |
| PedsQL self-report  Generic Total score  Generic PSHS  Generic Physical functioning  Generic Emotional functioning  Generic Social functioning  Generic School functioning  Cancer Pain | 39  39  52  52  50  39  56 | 65.27 (60.13 to 70.41)  70.86 (66.30 to 75.42)  50.74 (43.89 to 57.58)  71.34 (65.94 to 76.74)  80.57 (76.05 to 85.09)  58.71 (51.73 to 65.68)  60.78 (53.61 to 67.95) | 34  34  46  46  34  34  46 | 70.76 (65.32 to 76.21)  72.74 (67.91 to 77.57)  66.34 (59.14 to 73.55)  71.14 (65.45 to 76.83)  82.19 (77.49 to 86.89)  63.20 (55.81 to 70.59)  69.41 (61.61 to 77.20) | 34  34  35  35  35  34  37 | 75.21 (69.76 to 80.65)  77.05 (72.22 to 81.89)  70.45 (62.39 to 78.51)  78.58 (72.23 to 84.92)  82.51 (77.20 to 87.83)  69.38 (61.98 to 76.77)  70.26 (61.80 to 78.72) |

*CI: confidence interval; PedsQL: Pediatric Cancer Quality of Life Inventory™; PSHS: psychosocial health summary score*

**Supplemental Table S4**. Univariate differences in parent-reported HR-QoL between children with and without VIPN

|  | Generic Total score | | Generic PSHS | | Generic Physical functioning | | Generic Emotional functioning | | Generic Social functioning | | Generic School functioning | | Cancer Pain | |
| --- | --- | --- | --- | --- | --- | --- | --- | --- | --- | --- | --- | --- | --- | --- |
|  | **Beta (95% CI)** | **P value** | **Beta (95% CI)** | **P value** | **Beta (95% CI)** | **P value** | **Beta (95% CI)** | **P value** | **Beta (95% CI)** | **P value** | **Beta (95% CI)** | **P value** | **Beta (95% CI)** | **P value** |
| VIPN according to |  | | | | | | | | | | | | | |
| CTCAE or ped-mTNS | -10.64 (-16.41 to -4.87) | <0.001 | -7.34 (-12.62 to -2.06) | 0.007 | -16.03 (-22.63 to -9.43) | <0.001 | -6.30 (-10.87 to -1.73) | 0.007 | -3.48 (-8.30 to 1.34) | 0.16 | -6.72 (-15.33 to 1.88) | 0.12 | -13.17 (-19.45 to -6.88) | <0.001 |
| CTCAE | -12.41 (-20.19 to -4.63) | 0.002 | -9.48 (-16.52 to -2.45) | 0.009 | -17.58 (-25.49 to -9.67) | <0.001 | -9.65 (-14.96 to -4.34) | <0.001 | -4.12 (-9.85 to 1.60) | 0.16 | -9.87 (-21.16 to 1.41) | 0.09 | -14.80 (-22.24 to -7.37) | <0.001 |
| ped-mTNS | -11.70 (-17.82 to -5.57) | <0.001 | -7.84 (-13.24 to -2.43) | 0.005 | -15.13 (-23.44 to -6.82) | <0.001 | -3.25 (-9.11 to 2.61) | 0.27 | -5.65 (-11.24 to -0.07) | 0.05 | -8.68 (-17.27 to -0.08) | 0.05 | -15.79 (-24.19 to -7.39) | <0.001 |
| Severe VIPN according to |  | | | | | | | | | | | | | |
| CTCAE or ped-mTNS | -12.06 (-18.51 to -5.62) | <0.001 | -9.71 (-15.54 to -3.88) | 0.001 | -16.73 (-24.71 to -8.75) | <0.001 | -9.14 (-14.62 to -3.66) | 0.001 | -6.47 (-12.13 to -0.81) | 0.03 | -10.98 (-20.36 to -1.23) | 0.03 | -18.99 (-26.30 to -11.68) | <0.001 |
| CTCAE | -27.63 (-49.91 to -5.35) | 0.02 | -25.58 (-45.54 to -5.62) | 0.01 | -22.72 (-41.69 to -3.76) | 0.02 | -13.15 (-25.88 to -0.43) | 0.04 | 10.18 (-3.93 to 24.30) | 0.16 | -21.67 (-52.10 to 8.77) | 0.16 | -19.30 (-35.59 to -3.01) | 0.02 |
| ped-mTNS | -11.79 (-18.06 to -5.52) | <0.001 | -9.16 (-14.56 to -3.76) | 0.001 | -16.43 (-24.99 to -7.86) | <0.001 | -7.71 (-13.62 to -1.81) | 0.01 | -8.67 (-14.23 to -3.11) | 0.003 | -8.68 (-17.27 to -0.08) | 0.05 | -19.90 (-28.27 to -11.53) | <0.001 |

*PSHS: psychosocial health summary score, CI: confidence Interval, VIPN: vincristine induced peripheral neuropathy, CTCAE: common terminology criteria of adverse events, ped-mTNS: pediatric modified Total Neuropathy Score. No VIPN was used as reference category in all analyses.*

**Supplemental Table S5.** Univariate differences in self-reported HR-QoL between children with and without VIPN

|  | Generic Total score | | Generic PSHS | | Generic Physical functioning | | Generic Emotional functioning | | Generic Social functioning | | Generic School functioning | | Cancer Pain | |
| --- | --- | --- | --- | --- | --- | --- | --- | --- | --- | --- | --- | --- | --- | --- |
|  | **Beta (95% CI)** | **P value** | **Beta (95% CI)** | **P value** | **Beta (95% CI)** | **P value** | **Beta (95% CI)** | **P value** | **Beta (95% CI)** | **P value** | **Beta (95% CI)** | **P value** | **Beta (95% CI)** | **P value** |
| VIPN according to |  | | | | | | | | | | | | | |
| CTCAE or ped-mTNS | -8.96 (-14.61 to -3.31) | 0.002 | -4.03 (-9.09 to 1.03) | 0.12 | -17.62 (-25.60 to -9.65) | <0.001 | -8.84 (-14.88 to -2.81) | 0.004 | -3.31 (-8.55 to 1.93) | 0.21 | -1.68 (-9.68 to 6.31) | 0.68 | -21.75 (-29.33 to -14.17) | <0.001 |
| CTCAE | -9.47 (-17.25 to -1.71) | 0.02 | -5.88 (-12.70 to 0.94) | 0.09 | -12.78 (-23.40 to -2.16) | 0.02 | -8.10 (-15.89 to -0.30) | 0.04 | -5.10 (-11.64 to 1.51) | 0.13 | -6.21 (-16.85 to 4.42) | 0.25 | -29.10 (-38.70 to -19.46) | <0.001 |
| ped-mTNS | -12.32 (-17.89 to -6.75) | <0.001 | -7.12 (-12.21 to -2.04) | 0.007 | -20.90 (-28.98 to -12.82) | <0.001 | -9.86 (-16.26 to -3.46) | 0.003 | -5.88 (-11.25 -0.51) | 0.03 | -6.22 (-14.41 to 1.96) | 0.13 | -21.49 (-29.55 to -13.43) | <0.001 |
| Severe VIPN according to |  | | | | | | | | | | | | | |
| CTCAE or ped-mTNS | -11.66 (-17.48 to -5.85) | <0.001 | -8.01 (-13.19 to -2.82) | 0.003 | -18.19 (-26.48 to -9.90) | <0.001 | -11.17 (-17.36 to -4.97) | 0.001 | -5.14 (-10.54 to 0.27) | 0.06 | -10.41 (-18.56 to -2.27) | 0.01 | -21.90 (-30.11 to -13.70) | <0.001 |
| CTCAE | -12.29 (-34.54 to 9.95) | 0.28 | -9.30 (-28.54 to 9.95) | 0.34 | -23.13 (-48.08 to 1.82) | 0.07 | -13.67 (-32.06 to 4.71) | 0.14 | -10.41 (-25.42 to 4.61) | 0.17 | -23.94 (-53.47 to 5.59) | 0.11 | -30.24 (-59.59 to -0.90) | 0.04 |
| ped-mTNS | -13.12 (-18.81 to -7.43) | <0.001 | -9.24 (-14.35 to -4.12) | 0.001 | -19.97 (-28.26 to -11.68) | <0.001 | -11.35 (-17.79 to -4.92) | 0.001 | -6.54 (-11.97 to -1.12) | 0.02 | -12.77 (-20.76 to -4.77) | 0.002 | -22.99 (-31.31 to -14.67) | <0.001 |

*PSHS: psychosocial health summary score, CI: confidence Interval, VIPN: vincristine induced peripheral neuropathy, CTCAE: common terminology criteria of adverse events, ped-mTNS: pediatric modified Total Neuropathy Score. No VIPN was used as reference category in all analyses.*


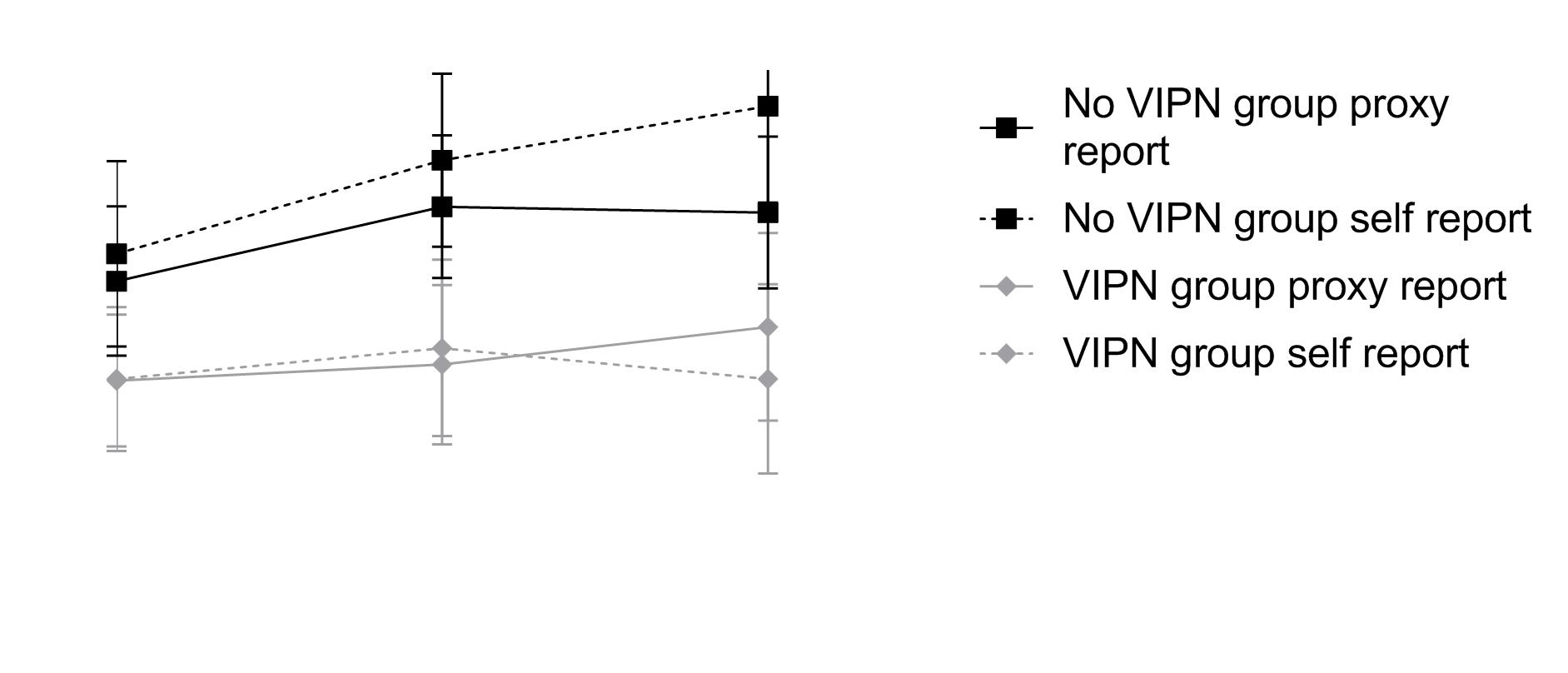

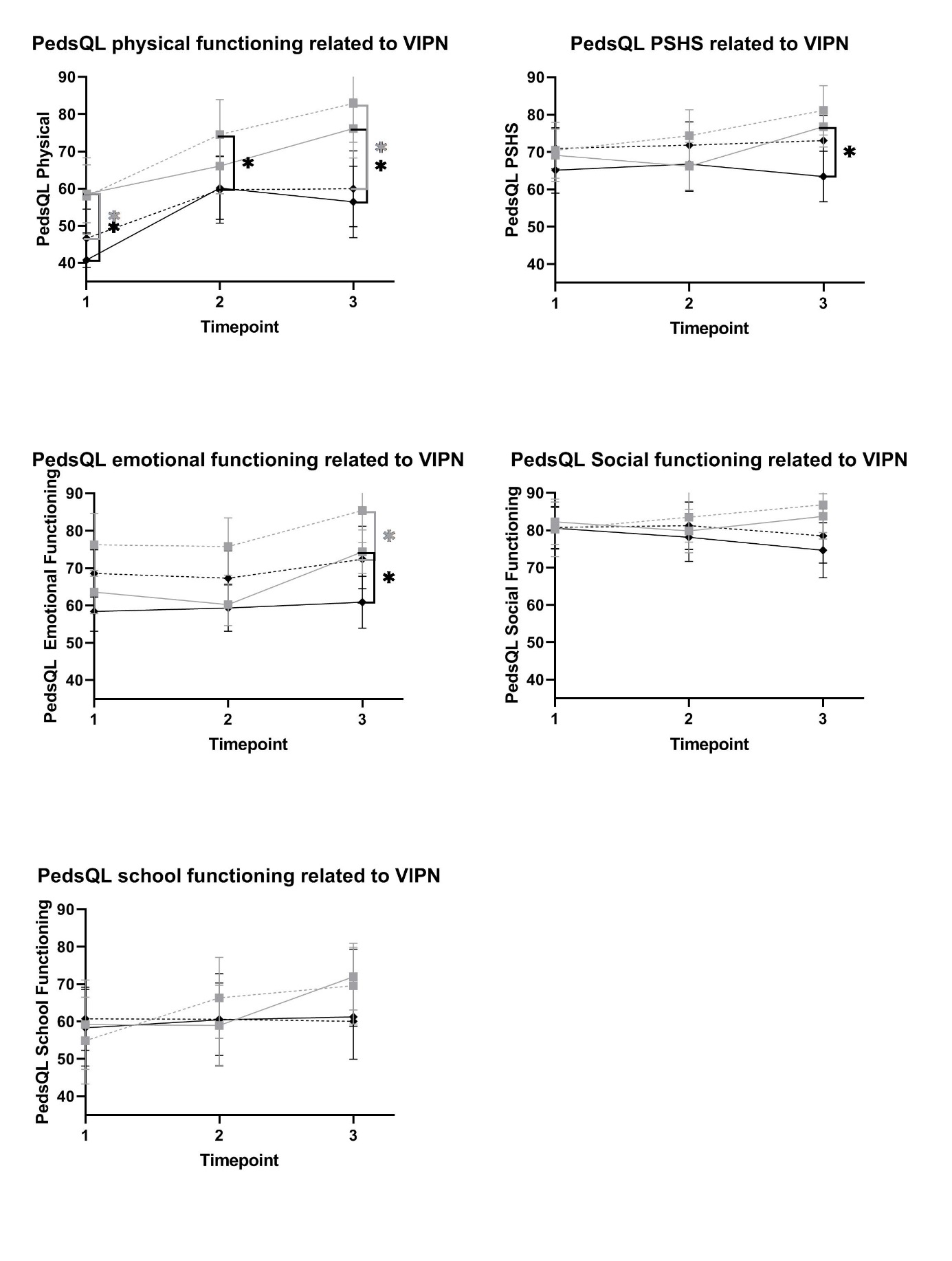
**Supplementary Figure S1.** PedsQL Generic scores divided in children with and without vincristine induced peripheral neuropathy according to either the Common Terminology Criteria for Adverse Events or pediatric modified Total Neuropathy Score

**represents p values < 0.05 per timepoint between the groups with and without VIPN, VIPN: vincristine induced peripheral neuropathy, PedsQL: Pediatric Quality of Life Inventory*

**Supplemental Figure S2:** Measurement schedule per treatment protocol
